# Speech and Language Therapists’ Views and Experiences of Working With People With Wernicke's Aphasia: A Qualitative Interview Study

**DOI:** 10.1111/1460-6984.70299

**Published:** 2026-07-25

**Authors:** Susanna Williams, Freyja Bell, Sarah Northcott, Suzanne Beeke

**Affiliations:** ^1^ East Suffolk and North Essex Foundation Trust Ipswich UK; ^2^ Department of Language & Cognition University College London London UK; ^3^ Croydon Health Services NHS Trust London UK; ^4^ Division of Language and Communication Science City St George's, University of London London UK

**Keywords:** communication partner training, multi‐disciplinary team working, therapeutic alliance, Wernicke's aphasia

## Abstract

**Background:**

Wernicke's aphasia is a challenging communication disability to live with, characterised by fluent speech, impaired auditory comprehension and reduced self‐monitoring. There is little literature addressing functional therapy approaches. For example, there is limited evidence for communication partner training (CPT) for this client group, despite research on its effectiveness more generally. It is not known how speech and language therapists (SLTs) understand and apply this limited and disparate evidence to their practice.

**Aims:**

To explore how SLTs assess and treat people with Wernicke's aphasia (PwWA), specifically how SLTs engage with PwWA; work with the multi‐disciplinary team to support PwWA; support significant others of PwWA, including through the provision of CPT.

**Methods:**

In this qualitative interview study, SLTs were recruited through social media and professional newsletters. Interviews via videoconferencing followed a topic guide and were transcribed and analysed using Framework Analysis. Fifteen SLTs participated, with 1.5–34 years of experience, working in England and Wales across a range of inpatient and community settings.

**Results:**

SLTs described a range of issues when working with PwWA. Building a therapeutic alliance was considered important, but could take longer than in other types of aphasia. Informal assessment was required for many PwWA, with published language assessments deemed unsuitable by many. It was perceived that available outcome measures did not capture change. SLTs took differing views on which theoretical models they should use to guide impairment‐based therapy. Treatment choices were sometimes driven by trial and error. SLTs offered CPT throughout the stroke pathway, reporting it to be clinically useful; however, there was uncertainty around which communication strategies to recommend. Many SLTs viewed working with PwWA negatively. They wanted to help but felt unsure about how to do so, and commented that the evidence base for treatment was limited. SLTs worked most closely with occupational therapists to manage Wernicke's aphasia. SLTs expressed concerns around the quality of decision‐making capacity assessments completed by other members of the multidisciplinary team and access to psychological support for PwWA.

**Conclusion:**

SLTs want evidence to guide their practice when working with Wernicke's aphasia. Further research is needed to inform CPT and outcome measures for PwWA. CPT should be supported by empirical research into interactions involving PwWA. There needs to be improved access to mental health support and high‐quality decision‐making capacity assessment for PwWA.

**WHAT THIS PAPER ADDS:**

*What is already known on this subject*

- Impairment‐based treatments for Wernicke's aphasia from varied theoretical perspectives are reported in the literature. There is little literature reporting on functional approaches and communication partner training for Wernicke's aphasia. It is not known how practising speech and language therapists (SLTs) assess and treat people with Wernicke's aphasia (PwWA).

*What this paper adds to existing knowledge*

- SLTs found PwWA difficult to work with; they wanted to help but felt unsure about what to offer. SLTs are offering communication partner training for Wernicke's aphasia throughout the stroke pathway; however, they expressed differing opinions on which communication strategies to recommend.

*What are the potential or actual clinical implications of this work?*

- SLTs would like more research evidence to guide them when managing Wernicke's aphasia. There is a need for assessments, treatments, outcome measures and psychological support that meet the unique needs of PwWA.

## 1 Introduction

### 1.1 Background

First described by Carl Wernicke in 1874, Wernicke's aphasia is characterised by impaired auditory comprehension, fluent speech and impaired repetition (Goodglass et al. 2001). It results in severe language impairment with jargon production and impaired self‐monitoring (Marshall 2008). Alternative terms include jargon aphasia (Marshall 2006) and fluent aphasia (Edwards 2005). Jargon aphasia is characterised by frequent neologisms; speech may or may not be fluent (Marshall 2006). Fluent aphasia is a broader term for all aphasia types where speech is fluent, both with comprehension impairments (Wernicke's aphasia, transcortical sensory aphasia) and without (anomia, conduction aphasia) (Edwards 2005). Following an acute stroke, Wernicke's aphasia accounts for 15% of all aphasia presentations; this reduces to 5% of presentations at 1‐year post‐stroke (Pedersen et al. 2004).

There is consensus that people with aphasia should receive an assessment from a speech and language therapist (SLT) to establish the nature, severity and consequences of communication impairment (Intercollegiate Stroke Working Party 2023; Simmons‐Mackie et al. 2017). Best practice is to offer personalised impairment‐based treatment and functional therapy targeting participation, including augmentative and alternative communication (AAC) and communication partner training (CPT) (Simmons‐Mackie et al. 2017).

For PwWA, impairment‐based approaches dominate the literature (e.g., Rogalski et al. 2013; Woodhead et al. 2017). Differing views on the theoretical basis for impairment‐based treatments include that Wernicke's aphasia results from a semantic impairment (Ellis et al. 1983), a phonological deficit (Pilkington et al. 2019), a semantic‐phonological impairment (Robson et al. 2012) or a semantic‐cognitive impairment (Wiener et al. 2004). Marshall et al. (1998) considered whether anosognosia, a lack of insight into neurological deficits, may play a role in monitoring failure, although this condition was considered to offer only a partial explanation. According to Marshall (2006), jargon may have communicative value. Some studies suggest there may be different types of Wernicke's aphasia (Ellis et al. 1983; Robson et al. 2012). It is not known how SLTs understand and apply theoretical perspectives to practice.

Single case studies reporting functional therapy approaches for Wernicke's aphasia target AAC and CPT. Maxwell et al. (2021) describes how a personalised word book was successfully used as low‐tech AAC alongside written word multiple choice. CPT research consists of case studies. Boles (2015) describes how CPT resulted in more balanced conversations. Blom Johansson et al. (2013) provided CPT and emotional support to the husband of a woman with severe acute Wernicke's aphasia, which, although valued, was unable to alter his belief that his wife had a memory problem, not a communication difficulty. There is a strong evidence base for CPT in aphasia (Simmons‐Mackie et al. 2010, 2016); however, only 16/56 studies included people with fluent aphasia, with some participant overlap across studies. Only one study reported on CPT in the acute stage for one person with Wernicke's aphasia (Blom Johansson et al. 2013). Further research into CPT with PwWA has been called for (Beeke et al. 2020; Marshall 2017).

To engage in speech and language therapy, people with aphasia need to build a reciprocal therapeutic alliance with their SLT (Lawton et al. 2018). However, comprehension difficulties and reduced insight can make it harder for SLTs to establish shared understanding and trust with PwWA, requiring greater flexibility to support engagement (Marshall 2008), and posing an additional barrier to forming an effective therapeutic alliance for people with this type of aphasia.

There is limited research on the ways in which SLTs work within the multi‐disciplinary team (MDT) to support PwWA, for example during decision‐making capacity assessments (Maxwell et al. 2021), and to enhance communication, for example between nurses and PwWA (Hersh et al. 2016). However, speech and language therapy rehabilitation was not the focus of these studies. Given the challenges that PwWA experience in comprehension, insight, developing a therapeutic alliance, and engaging in therapy (Marshall 2008), it is important to understand current speech and language therapy practice regarding Wernicke's aphasia.

### 1.2 Aims

The aim of this study was to explore how SLTs assess and treat PwWA, specifically how SLTs: engage with PwWA; work with the MDT to support PwWA; support significant others of PwWA, including through the provision of CPT. The objective is to inform future research, service and policy development.

## 2 Methods

In this qualitative study, SLTs completed in‐depth interviews via videoconferencing. Reporting follows the Consolidated Criteria for Reporting Qualitative Research (COREQ) (Tong et al. 2007) (Supporting Information 1). Ethical approval was granted by the University College London Department of Language and Cognition ethics committee, reference LCD‐2023‐04. All participants gave written informed consent. Names and identifying details have been changed to preserve anonymity.

### 2.1 Recruitment and Sampling

UK‐based SLTs working in the National Health Service (NHS) and independent practice were eligible to take part, provided they had worked with at least two PwWA since qualifying, one in the last year. The study was promoted via X (formerly Twitter), the UK Stroke Forum 2023, and through the British Aphasiology Society, the Council for Allied Health Professionals in Research and the Association of Speech and Language Therapists in Independent Practice. Participants received a £10 electronic book voucher for participating. Convenience sampling permitted data collection within the available time.

### 2.2 Participants

Thirty SLTs expressed an interest and were provided with a participant information sheet. The first 15 SLTs who responded and arranged an interview were recruited. This was a pragmatic decision that allowed a diverse range of views to be captured within the timeframe available for the study. Participants provided demographic information via an online form. SLTs from across England and Wales took part. All participants were female. The majority were white British (73%). Participants had worked as an SLT for between 1.5 and 34 years. Eleven participants worked in more than one setting. For participant details, see Table 1.

**TABLE 1:** Participants details (*n* = 15).

Characteristic	*n* (%)
Age (years)	
20–29	2 (13%)
30–39	6 (40%)
40–49	2 (13%)
50–59	4 (27%)
60 or older	1 (7%)
Ethnicity	
White British	11 (73%)
White non‐British	3 (20%)
Did not state	1 (7%)
Number of years working as an SLT	
1–5	2 (13%)
6–10	4 (27%)
10–20	3 (20%)
20+	6 (40%)
Current setting(s) in which participants treat people with Wernicke's aphasia	
Community stroke and neurological rehabilitation teams	5
Early Supported Discharge Team	5
Hyper‐acute and stroke units	5
Stroke rehabilitation unit	5
Private practice	3
Specialist neurological rehabilitation unit	3
Community speech and language therapy team	1
Charity providing community support	1
Region in which participant works	
East Midlands	2
East of England	1
Greater London	3
North West England	2
South East England	3
South West England	1
Wales	1
West Midlands	1
Yorkshire and The Humber	1

*Note*: 11 participants reported working in more than one setting.

### 2.3 Topic Guide

Interviews followed a topic guide (Supporting Information 2) developed with stakeholder involvement. It was initially drafted by S.W., a specialist SLT in acute stroke, to reflect research aims, published texts offering an overview of assessment and treatment for Wernicke's aphasia (Marshall 2008) and jargon aphasia (Marshall 2017), studies of speech and language therapy practice (e.g., Borrett and Gould 2020) and personal clinical experience. The topic guide was reviewed by co‐authors, and a practising SLT and experienced stroke occupational therapist (OT), who were colleagues of S.W. It was also discussed at a meeting of the British Aphasiology Society with 25 SLT attendees. The topic guide was amended based on stakeholder feedback. For example, at the suggestion of the OT, ‘MDT understanding of Wernicke's aphasia’ was added to a general ‘MDT working’ item. Participants were asked to describe a time when assessing and treating a PwWA went well, and another time when there were challenges. The best term for the targeted aphasia type was debated within the study team and with stakeholders. The term Wernicke's aphasia was selected on the basis that it is succinct and recognisable. The characteristics of Wernicke's aphasia were described in the participant information sheet and at the start of the interview, so that participants were fully aware of the aphasia type under discussion.

### 2.4 Data Collection and Transcription

Participants took part in in‐depth semi‐structured recorded interviews using Microsoft Teams. Interviews took place between December 2023 and February 2024, were conducted by S.W., and lasted, on average 49 min (ranging from 33 to 59 min). Participants reflected on their experience of assessing and treating people with Wernicke's aphasia.

The topic guide was used flexibly. Probing questions enabled a more in‐depth understanding of participant views and experiences. All participants were asked to discuss two cases in detail, one where they felt that assessing and treating someone with Wernicke's aphasia went well, and another where there were challenges. This approach was chosen to elicit a detailed description of practice and facilitate reflection. Of the 15 SLTs who participated, 14 shared two cases. One participant was unable to identify a case where they felt assessment and treatment went well, so they only shared a case that they found challenging. The study team met to review progress after five interviews had been completed. It was noted that participants used the terms fluent aphasia, jargon aphasia and Wernicke's aphasia. As a result, the topic guide was amended to explicitly ask participants how they would label the type of aphasia being discussed.

Interviews were automatically transcribed by Microsoft Teams, and any errors were corrected by S.W., who also anonymised the transcripts by changing names, places and potentially identifying details. Video recordings were then deleted.

### 2.5 Data Analysis

Data analysis was completed by S.W. using Framework Analysis (Ritchie and Spencer 1994), a matrix‐based approach that allowed for systematic analysis of the range of views and experiences within themes and across participants, exploring common features and differences, and relationships between views and experiences. NVivo version 14 and Excel were used to manage the data. Initial themes were identified through immersion and used to construct a thematic index (Supporting information 3). The index was applied to the transcripts, with every phrase or passage assigned a code. Thematic charts were created; chart headings were developed during the indexing process. The indexed data were summarised into matrices.

### 2.6 Trustworthiness and Bias

S.W. and F.B. are clinical‐academic specialist SLTs with experience of working in acute stroke units, Early Supported Discharge services, and community speech and language therapy teams. S.N. and S.B. are experienced qualitative researchers and academic SLTs. Participants were aware of S.W.’s professional background. S.W. maintained a neutral stance during interviews by asking questions to encourage rich descriptions without expressing her own views. Member checking was completed throughout the interviews with S.W., who regularly summarised the participants’ responses and invited correction or elaboration. S.W. kept reflective research notes throughout the study design, data collection and analysis phases, enabling her to reflect on any conscious or unconscious bias.

The study team met regularly during data analysis to support interpretation. F.B. independently reviewed three transcripts to determine that the index captured the themes identified in the data. She reviewed the charts to see that themes were fairly reflected. S.N. and S.B. read two transcripts and reviewed the thematic index and charts, and further refinements to the themes were made.

## 3 Results

Six themes were identified from the interviews: (1) SLTs’ knowledge, skills and attitudes towards working with PwWA; (2) identifying and labelling Wernicke's aphasia; (3) engaging PwWA in speech and language therapy; (4) assessing aphasia, insight and outcomes; (5) treatment approaches and (6) MDT working and Wernicke's aphasia. Supporting quotes from participants are provided and identified by participant number after a quote.

### 3.1 SLTs’ Knowledge, Skills and Attitudes Towards Working With People With Wernicke's Aphasia

Working with PwWA evoked a range of largely negative emotions in SLTs. On meeting PwWA, SLTs described feelings of panic and dread ‘my heart always sinks a little bit’ (P1). Wernicke's aphasia was considered ‘scary’ (P3) and ‘uncomfortable’ (P10) to work with. Wernicke's aphasia was thought to be more ‘stubborn’ (P7) to treat than other types of aphasia. SLTs reported feeling frightened when working with PwWA who were agitated and lacked insight. P12 recalled ‘feeling quite intimidated by him because he was quite a tall, large gentleman. And he'd get really agitated if I tried to challenge him on anything’. A less common perspective was that SLTs enjoyed working with PwWA and found them ‘fascinating’ (P4).

SLTs wanted to help PwWA but largely felt that they did not know how to do so, ‘I don't get many patients … but when we get them, we don't have a clue what we do’ (P9). Less commonly, SLTs found treatment to be straightforward. This occurred when goals were functional rather than linguistic, and the PwWA had a family member who accepted their communication difficulties and was willing to adapt their own communication. SLTs were aware of the impact of their negative perceptions on PwWA. P3 said ‘I think that's [the SLT's negative perception] a challenge for the poor person with fluent aphasia’. SLTs expressed high levels of concern and empathy for PwWA, for example, P7 described how ‘I really empathise for inpatients with communication difficulties, because they must have a really rough trot [experience]’. SLTs considered PwWA to be vulnerable, ‘They are the walking wounded. But there's also a huge vulnerability for people with that type of language impairment’ (P8).

Treating Wernicke's aphasia for the first time acted as a catalyst for SLTs to develop their knowledge and skills. P8 described learning ‘from my first patient. He taught me so much’. Wernicke's aphasia was most likely taught variably at universities, and newly qualified SLTs reported that upon graduation they were able to identify Wernicke's aphasia but had limited knowledge of treatment options. This view was shared by SLTs supervising newly qualified SLTs. Post‐qualification, SLTs developed their knowledge and skills through reading, shadowing colleagues, experience and reflective practice. P13 described having ‘learnt the hard way, at the expense of people that I potentially could have helped’. SLTs wanted more information about Wernicke's aphasia but were limited by a lack of published research specifically targeting Wernicke's aphasia, ‘I'm not sure there is stacks that has come up about fluent aphasia … they're a neglected group of people’ (P3).

### 3.2 Identifying and Labelling Wernicke's Aphasia

SLTs viewed identifying Wernicke's aphasia as an important part of their role, however, they depended upon others recognising that the person had communication difficulties and that SLT input was needed. SLTs were concerned that Wernicke's aphasia was not always recognised as a symptom of stroke. ‘People behave oddly. They might be quite aggressive … and then they start talking incoherently. The first thing people think of is calling the police, isn't it? And then they get sectioned’ (P13). In hospital, they may be misdiagnosed as being ‘confused’ (P11).

To SLTs, the diagnosis of Wernicke's aphasia was usually obvious: ‘She fell into that box’ (P11). SLTs knew the characteristics of Wernicke's aphasia and felt that information describing it was easily accessible to them. SLTs based their initial diagnostic impression upon the person having comprehension difficulties that were worse than their expressive language, fluent speech containing jargon words, phonemic and semantic errors. P2 described a person she treated who ‘presented quite typically with Wernicke's …Expressive was relatively better. Fluent. Just a lot of jargon and a lot of phonemic errors’. PwWA were largely described as having mild or no physical impairments. SLTs reported that exceptionally PwWA presented with more significant limb weakness, dysphagia, memory problems and behavioural problems.

SLTs used different terms to describe Wernicke's aphasia for different purposes and audiences. *Fluent aphasia* was used by 80% (12/15) of participants, *Wernicke's aphasia* by 53% (8/15) and *jargon aphasia* by 27% (4/15). *Fluent aphasia* was widely used with PwWA, their families and SLTs. SLTs liked this term because it was descriptive, but they were aware that technically it includes people with good auditory comprehension, whom they intended to exclude from this label. SLTs used *Wernicke's aphasia* with doctors, nurses and SLTs. They felt that ‘you can just say Wernicke's and people know straight away what you're talking about’ (P13) but that with ‘localisation theory becoming more in doubt’ (P13) some had moved away from this label. SLTs did not always specify which type of aphasia people have when speaking to PwWA or their families to avoid making the information they shared too ‘complex’ (P15).

### 3.3 Engaging PwWA in Speech and Language Therapy

SLTs engaged PwWA in therapy by building a therapeutic alliance and highlighting their communicative competence. They did so by spending time listening, discussing topics of interest to the individual, facilitating communication with others, and using humour. P4 described how she worked collaboratively with PwWA because ‘I think people often treat them like they're incompetent communicators’. SLTs accepted that building engagement could take several weeks, ‘it actually took him a long time to engage in therapy … but once he did start engaging it was amazing’ (P8). Having ‘buy in from a familiar communication partner, somebody who the person with aphasia relies on and trusts … a family member’ (P7) positively influenced engagement.

Barriers to engagement included PwWA lacking insight into their communication difficulties, ‘he didn't understand why he needed to do it’ (P12), severe receptive language impairment and cognitive impairment, particularly attention difficulties. SLTs found it challenging to engage PwWA when they did not want to be in hospital. SLTs reported using a range of strategies when PwWA did not engage in therapy sessions, including allowing more time for spontaneous recovery, continuing to build a therapeutic alliance and returning ‘back to that goal setting and what you're trying to achieve’ (P1). An alternative approach was to ‘set up their communication environment to just be a bit more supportive’ (P14) by offering education and CPT to others.

SLTs valued clear goals but reported that it was challenging to involve PwWA in the goal‐setting process. The expressive and receptive language impairments associated with Wernicke's aphasia were barriers to goal setting, ‘their receptive language was really impaired …and no insight. So, it was really hard to figure out goals and how to move forward’ (P2). Rehabilitation services were particularly goal‐oriented, and identifiable goals were required for transfer from an acute stroke unit to an inpatient rehabilitation unit. SLTs felt that functional goals were most achievable, for example, ‘to go to the cafe again with his wife’ (P14). Goals for PwWA and their communication partners were considered highly relevant when they ‘focused on improving quality of life and … what are these frustrations [with communication] and what can we do to kind of ease that on both sides?’ (P9).

### 3.4 Assessing Aphasia, Insight and Outcomes

Comprehensive assessment was considered important for PwWA. Many SLTs felt that formal language assessments were unsuitable for PwWA, ‘the sort of assessments that we have available are just not fit for purpose for Wernicke's … they're not constructive, they don't lead me anywhere’ (P5). SLTs reported that completing formal assessments could be ‘stressful’ (P10) and ‘tricky’ (P2). Difficulties with attention and insight were barriers to completing formal assessment for some PwWA. Comprehension difficulties prevented PwWA from understanding assessment instructions. High levels of failure during formal assessment resulted in frustration and distress. SLTs observed the experience to be ‘demoralising’ (P5) and ‘invalidating’ (P4) for some PwWA. Alternatively, other PwWA tolerated formal assessment well.

SLTs used a combination of informal assessment and observation to evaluate features unique to Wernicke's aphasia. PwWA were described as ‘masking’ (P8) their comprehension difficulties by using non‐verbal cues to aid their understanding and by holding the communicative floor. SLTs noted that PwWA appeared to have better auditory comprehension in conversation than when tested, ‘couldn't do a direct command … but socially did incredibly well’ (P10). SLTs observed the positive effect of context upon the expressive language of PwWA, ‘was his speech more coherent … when in context? Absolutely’ (P10). Informal reading assessment was valued by SLTs. P3 reported ‘it feels very important to establish what their written word understanding at single word level is because that is so useful for them’.

Insight was assessed based upon behavioural observations. Having no insight was characterised as being unaware of speech errors, blaming communication difficulties on other people, and denying that there is a communication impairment, ‘they're quite cross because they don't have insight, they just think everyone else is just having a laugh … “Why can't you understand me? This is ridiculous!”’ (P4). Exceptionally, PwWA were described as ‘blissfully unaware’ (P11). Having ‘extremely good insight’ (P3) was characterised by the use of total communication strategies, having some self‐monitoring ability and showing embarrassment about their communication difficulties.

SLTs reported outcomes for PwWA mainly in descriptive terms and the use of quantifiable outcome measures was an exception. SLTs described how PwWA demonstrated improvements in auditory comprehension and expressive language, especially a reduction in jargon. They also described improvements in function, mood and quality of life. Available outcome measures were felt to be too generic and did not capture change for PwWA. P3 described how, when she worked with the daughter of a woman with Wernicke's aphasia, she ‘made a rough assessment of what percentage of her spoken output in conversation was accurate and appropriate at the beginning of my intervention, that we were agreed on, and we made one at the end’. SLTs felt that when working with PwWA it was necessary to have a ‘different understanding of what success is’ (P5) than when working with people with other types of aphasia. P5 reflected on discharging a woman with chronic Wernicke's aphasia who had returned to her own home after living in a care home and ‘wasn't massively frustrated by it [aphasia] … so it wasn't impacting her life’. Despite this positive outcome, P5 reflected ‘I think that as a speech therapist, you always hope that you're going to have more obvious outcomes’.

### 3.5 Treatment Approaches

There was widespread agreement that PwWA should be offered early speech and language therapy. Where PwWA were unable to engage in early therapy due to difficulties with insight, SLTs worked on insight building. SLTs raised the insight of PwWA through education, therapy tasks, video feedback and immediate feedback when their speech did not make sense. PwWA received SLT input in hospital and immediately following discharge through Early Supported Discharge (ESD) teams and community neurological teams. They were referred on to community SLT teams unless the person with aphasia and their family did not want further input, ‘They didn't want any more from us. I think they were as sick of us, as we were of trying to help’ (P10). The majority of SLTs working with chronic Wernicke's aphasia in both NHS and private settings felt that intervention was no longer appropriate 1–2 years post‐stroke on the basis that no further language recovery is possible for PwWA, and that this is sufficient time for individuals and their families to adjust, ‘I don't generally feel that I can make an awful lot of difference at that point’ (P13). SLTs referred PwWA to community aphasia groups if available but expressed mixed views about whether they were suitable. SLTs felt that small, SLT‐led groups best met the needs of PwWA. Meeting other PwWA was considered helpful as it ‘takes away some of the distress about the impairment’ (P3). SLTs reported that it was not common for PwWA to attend communication groups on a long‐term basis because PwWA were aware of ‘how they were perceived by other people’ (P3).

### 3.6 Impairment‐Based Therapy

There was disagreement about whether to offer an impairment‐based approach, a functional approach, or to take a ‘dual approach’ (P6) working in both ways at the same time. Additionally, SLTs described treatment choice as being driven by ‘trial and error’ (P5) or a ‘scattergun’ (P3) approach. Impairment‐based approaches were offered to specific PwWA who SLTs felt would benefit. Not all SLTs felt impairment‐based approaches were beneficial. SLTs who preferred a functional approach felt that it would result in the greatest change, ‘at the end of the day, is this person going to make significant progress on an impairment level? And if not, what can we do to improve quality of life more generally?’ (P11). Those favouring a dual approach found impairment‐based and functional approaches to be complementary, they valued ‘communication partner training and that kind of dual approach targeting their functional communication and communication success. And then obviously, you can do your traditional aphasia therapy as well’ (P7).

When SLTs offered impairment‐based therapy, the most common view was that auditory comprehension should be worked upon first, on the basis that ‘potentially we could have a bit of a double whammy, we could improve her auditory comprehension, but also we could improve her output if she could monitor what she was saying a bit more successfully’ (P4). SLTs would ‘inhibit the flow’ (P3) of speech to work on auditory comprehension tasks, ‘I spend a lot of time just going “No, we're not talking. We're just listening”’ (P6). When it was not possible to work on auditory comprehension due to attention difficulties, SLTs targeted non‐verbal attention first, for example, completing a ‘colouring sorting task for five minutes’ (P12). An alternative view was that auditory comprehension could improve indirectly when semantics were targeted, ‘that's gonna sort itself out by the context within that activity … there's no point in trying to throw in spoken information at people because they don't get it’ (P13). Difficulties with auditory comprehension and insight were reported as barriers to completing impairment‐based tasks with PwWA, resulting in a lack of understanding of task instructions and purpose. SLTs used demonstration to compensate for this, ‘as much demonstration as possible, as little [SLT] talking as possible’ (P13).

SLTs expressed five distinct views on the theoretical basis for Wernicke's aphasia (see Table 2). One was that Wernicke's aphasia is an intertwined cognitive and language impairment. SLTs who disagreed with this position felt that this was a misperception which negatively impacts the therapy that PwWA receive. Alternative views were that Wernicke's aphasia is a deficit of semantics, phonology or both. Another proposition was that there are a range of Wernicke's profiles without a single theoretical basis. SLTs expressed considerable uncertainty about the theoretical basis underpinning treatment of Wernicke's aphasia and when discussing case studies, they described changing their views over time across different clients.

**TABLE 2:** SLTs’ views on the theoretical basis for Wernicke's aphasia.

Theoretical basis	Quotes
A cognitive‐linguistic impairment	‘She ended up again with fluent aphasia. And that was really her only thing other than obviously the cognitive impairments that are related to that’ (P15).
A semantic deficit	‘Semantics is where it begins and ends for me. If they've got rubbish semantics, there's no point in working on anything else until you've sorted everything else out. If they've got good semantics, you know how to facilitate the output’ (P13).
A phonological deficit	‘I learned a bit more about that phonological stuff, and the fact that the semantics stuff is not very helpful’ (P10).
A semantic and phonological deficit	‘I think something I personally notice in a lot of our Band 5's is their kind of lack of awareness of phonological processing. There's often this huge focus on semantic stuff, which is still just as important, but quite often, and particularly in this kind of client group, it's overlooked massively’ (P15).
A range of Wernicke's profiles	‘She was the type of Wernicke's where it was very clear that her cognition was quite good. I don't know if you can say that fairly, when somebody's so aphasic. But … you get a feel when you work with different people, with different Wernicke's profiles’ (P7).

### 3.7 Communication Partner Training

SLTs believed that CPT was beneficial for families of PwWA. P7 said ‘I think if there's anyone you're gonna be involved with the communication partners, it's somebody with a Wernicke's’. CPT sessions were offered informally without following a specific programme. SLTs widely felt that there was a lack of suitable therapy materials and of evidence supporting the use of CPT for PwWA. CPT was delivered on an ad hoc basis alongside other SLT interventions. It was common for SLTs working in stroke units and ESD services to offer CPT shortly after PwWA had had their stroke, and when preparing for discharge. A less common view was that it was more appropriate to provide CPT 3 months or more post‐stroke once a family have had ‘a bit of time to actually accept that it's not going to get better’ (P6).

Identifying effective communication strategies was considered by SLTs to be an important part of their role, ‘I think that was the main thing that was really helpful was coming up with strategies to support her communication’ (P2). At times, SLTs prioritised this over impairment‐based therapy. Identifying communication strategies was described as an ongoing process throughout assessment and treatment. Communication strategies were identified through trial and error and based upon knowledge of the individual. SLTs recommended PwWA and their communication partners to use total communication strategies (e.g., to support comprehension ‘Write it down. Point. Show her’ (P3)).

SLTs held varied opinions on the best communication strategies for Wernicke's aphasia. Firstly, SLTs held different views on whether comprehension was aided by providing more or less information. A commonly held position was that communication partners should use simple language. P14 described advising a family member of a man with Wernicke's aphasia about ‘Reducing your own level of communication. Not giving him too much information’. An alternative perspective was that giving more information was helpful, ‘With a Broca's aphasia you would modify language. You use one word at a time, but you kind of do the opposite almost with Wernicke's. The more information, the more they seem to process and get what you're talking about’ (P1). Secondly, contrasting perspectives were evident concerning whether PwWA should be encouraged or discouraged from speaking at length. SLTs suggested allowing PwWA to take longer conversational turns so that others could listen for ‘the gist’ (P3) of what was being said, rather than focussing on the communication difficulties. P13 said ‘He was amazing in the stuff that he came out with. That you could get, just from sitting back and listening. He could tell you really complicated stuff’. Less commonly, SLTs recommended that family, friends and healthcare professionals should tell PwWA when they are not making sense, ‘I would say to the daughter … “You are going to have to stop her”. And the daughter said, “It's hard for me to stop her”. But the family were doing a lot of just nodding along and then realising “Well, we've lost it”’ (P3). P14 described how they asked the wife of a man with Wernicke's aphasia to ‘indicate fairly early on, “I'm sorry, it's not making sense. Is this to do with?”’ The SLT reported that this enabled the man to gain insight but was uncomfortable to watch.

During CPT sessions, SLTs offered education about stroke, Wernicke's aphasia and prognosis to the family of PwWA. SLTs described challenges when explaining Wernicke's aphasia, ‘I think it's such a strange communication disorder that it's hard to explain in a way that people really understand it’ (P9). Misconceptions shared with SLTs by family included that they thought the person with Wernicke's aphasia ‘wasn't trying’ (P2) or ‘was doing it on purpose’ (P9). Other family members took everything that PwWA said ‘at face value’ (P13). SLTs attributed the problems that family members had in appreciating comprehension difficulties to the fact that PwWA were masking.

### 3.8 Augmentative and Alternative Communication

SLTs provided PwWA with low‐tech AAC aids, including communication charts, books and cards. Highly personalised communication books were accepted by PwWA who were able to use compensatory communication strategies. P4 described a woman with Wernicke's aphasia who used a notebook to support her communication, ‘She will now push the book across to me so that I can write something down for her, or she'll have a go. She'll go “Oh no!” And then she'll write something’. Most PwWA preferred to communicate verbally, but some individuals accepted communication books as a means to get what they wanted, ‘I wouldn't say he was problem solving, that he was having a communication breakdown … I think he just learned that “I'll get my book out because that's what we've practiced”’ (P14). When PwWA lacked insight into their communication difficulties, AAC use did not generalise ‘He would reluctantly do it [use AAC] in therapy, but he'd already said what he wanted. So, he couldn't really see the point of like, “Why do I have to point to the picture as well?”’ (P12). Family members of PwWA used AAC to deal with misunderstandings in conversations. P10 provided a communication book to a woman with Wernicke's aphasia whose family were ‘Very much using it [AAC] as a tool for them. So, she wouldn't naturally use it, but they'd be like “Can you show me who you're talking about? Like, can you show? Is that what you want?”’

### 3.9 Multi‐Disciplinary Team Working and Wernicke's Aphasia

SLTs worked most closely with OTs, doing joint cognitive assessment, cognitive rehabilitation and discharge planning for PwWA. OTs and clinical psychologists attempted formal cognitive assessments with some PwWA, however these were not successful and informal assessment (e.g., of attention, executive function) through observation of functional activities was required. SLTs reported that other members of the MDT found it challenging to attribute whether difficulties observed during assessments were due to cognitive impairment or receptive language difficulties without joint working, ‘OT felt like they couldn't … figure out what was going on cognitively versus what was just receptive’ (P2).

SLTs advocated for PwWA to receive treatment from mental health professionals in the MDT, when needs were identified, and supported individuals’ access to this. SLTs reported that PwWA experienced frustration, agitation, low mood, difficulties adjusting to their disability, anxiety, relationship problems and suicide attempts. SLTs working in post‐acute services reported addressing mental wellbeing by working indirectly alongside psychologists to enable PwWA to access their services, providing psychological support themselves and referring PwWA to private dual‐trained SLT‐counsellors that they knew personally. P13 felt that ‘if the impairment's quite severe then I don't find straightforward counselling or psychology often that helpful. You need somebody who's got a specialism in aphasia before you can really make a difference’. SLTs working in acute settings did not report addressing mental wellbeing.

A range of concerns were expressed about MDT practice around mental capacity assessment regarding discharge decisions and medical procedures. Mental capacity assessments were conducted jointly by OTs, psychologists and doctors, with SLTs facilitating communication. Doctors were also reported to conduct mental capacity assessments alone with PwWA. In these instances, SLTs shared concerns that doctors may not have sufficiently appreciated the extent of the individual's communication difficulties or adapted their communication sufficiently to support understanding. P8 reported that when a man with Wernicke's aphasia was readmitted to hospital ‘the doctors didn't know how to communicate with him, and he ended up having his entire leg amputated and we don't think they got informed consent to do that’. PwWA were ‘often left out of decision making. It's often assumed that they lack capacity’ (P4).

## 4 Discussion

Fifteen SLTs completed in‐depth interviews exploring their experiences of assessing and treating PwWA. They described multiple issues resulting from the comprehension impairment and reduced insight associated with Wernicke's aphasia, confirming Marshall's (2008) assertion that a different approach to clinical management is required for patients with this type of aphasia. SLTs expressed different views regarding the theoretical frameworks used to guide impairment‐based therapy and highlighted limitations in the current evidence base. Blom Johansson et al. (2013) identified the need for emotional support of carers of people with newly diagnosed Wernicke's aphasia. SLTs in this study reported a range of mental health difficulties experienced by PwWA and suggested that improvements are needed in the provision of mental health support.

SLTs described delivering CPT to PwWA and their family carers in the days and weeks following stroke, while the evidence base for CPT is largely based upon studies involving people with chronic aphasia (Simmons‐Mackie et al. 2016). The limited evidence base for CPT in the acute stage of Wernicke's aphasia, as noted by SLTs in this study, reflects a significant gap in the literature. Nonetheless, SLTs view CPT as a valuable clinical tool, highlighting the need for further research.

SLTs felt it was important to advise on the best strategies to support comprehension and expression for PwWA but expressed uncertainty and differing views about which ones to recommend. When supporting comprehension in Wernicke's aphasia, SLTs had different views about whether to provide more or less verbal information. SLTs described PwWA as ‘masking’ their auditory comprehension difficulties by using non‐verbal cues; however, speakers without aphasia make use of non‐verbal cues to aid comprehension (Opoku‐Baah et al. 2021). When supporting expression, some SLTs suggested encouraging PwWA to take longer turns while a communication partner listens for the ‘gist’, while others suggested stopping the PwWA when their speech was not making sense. It is possible that all strategies described might be appropriate for particular PwWA and their communication partners at particular times. Empirical research that could enable SLTs to understand interactions involving PwWA is limited. Beeke et al. (2020), in their conversation‐analytic study of Wernicke's aphasia, found repair strategies in use that are considered to be interactionally dispreferred (and thus uncommon) in typical conversations, for example, correcting speech errors. Correcting the speech of another person usually reflects an interactional imbalance, for example, between a teacher and a pupil. In Beeke et al. (2020) data, these corrections were not treated by the woman with chronic Wernicke's aphasia as a threat to her competence as a communicator. This highlights the potential for different communication strategies to be relevant in conversations between PwWA and their communication partners than those that have been researched for other aphasia types.

SLTs expressed considerable concerns regarding MDT practice when assessing decision‐making capacity in relation to PwWA. Healthcare professionals did not always appreciate the extent of the communication difficulties experienced by PwWA and failed to adapt their own communication sufficiently. This is in keeping with Jayes et al.'s (2020) systematic review finding that people with communication difficulties do not always receive appropriate communication support during capacity assessments. In Maxwell et al.'s (2021) case study, joint SLT‐OT working was key to revealing decision‐making capacity for an individual with Wernicke's aphasia. Maxwell et al. (2021) used supported conversation, highly personalised AAC, and assessment of functional capacity through everyday tasks, which were strategies suggested by SLTs in this study.

SLTs reported challenges in measuring therapy outcomes for PwWA, and the majority of SLTs working with chronic Wernicke's aphasia were not sure speech and language therapy 1–2 years post‐stroke was beneficial. They referred PwWA to community stroke groups despite doubts about their suitability. These views are consistent with those reported in Hersh's (2001) case study about a man with fluent aphasia who was discharged when his SLT felt that ‘there was not much more that she could offer’ and referred to a group despite not being keen to attend. According to Bakheit et al. (2007), when outcomes were formally assessed 24 weeks post‐stroke, PwWA appeared to have a poorer prognosis than those with other aphasia types. However, when functional language assessments were reported (Laska et al. 2001) or longer follow‐up periods were considered (Kertesz and McCabe 1977), PwWA demonstrated improvements. The UK and Ireland National Clinical Guideline for Stroke (Intercollegiate Stroke Working Party 2023) describes how the term ‘rehabilitation potential’ has been used by clinicians to justify not providing a service. The guideline recommends that clinicians take a broader view of rehabilitation to include psychological wellbeing and social participation. Challenges in identifying which outcomes to measure and how may have shaped SLTs’ view that PwWA may not benefit from speech and language therapy in the chronic stage. While Marshall (2008) suggested that alternative outcome measures such as ratings of interaction success should be used for PwWA, it was uncommon for SLTs to use these.

### 4.1 Implications for Clinical Practice

Clinical supervisors of newly qualified SLTs may wish to consider that SLTs found treating PwWA for the first time particularly challenging. Offering additional support, such as opportunities to shadow experienced clinicians and to discuss PwWA on their caseload, could improve SLTs’ satisfaction when working with Wernicke's aphasia. Enhancing communication training for healthcare professionals to improve knowledge of the unique features of Wernicke's aphasia and skills in facilitating communication for PwWA would result in improvements in diagnosis, in the quality of assessments of decision‐making capacity, and in the provision of mental health support.

### 4.2 Strengths, Limitations and Future Directions

This study captured the views of SLTs working with varying levels of experience and working in a diverse range of settings. Reflexivity was actively prompted through the use of multiple analysts. SLTs were required to recall past times when they had worked with PwWA and may have been subject to recall bias. The lack of cultural and linguistic diversity in the SLT sample may have affected reported practices. Future research should seek the views of a diverse professional population. The variety of overlapping diagnostic labels applied by SLTs (*fluent aphasia*, *Wernicke's aphasia*, *jargon aphasia*) presented a challenge when researching this population. The heterogeneity of post‐stroke aphasia is well known, and the benefits of classifying aphasia have been questioned (Marshall 2010). In future research, using a longer descriptive label such as *fluent aphasia with comprehension impairments* could more clearly identify the population of interest to practising SLTs.

The findings of this study support further research into speech and language therapy assessment and treatment for PwWA. SLTs used varied treatment approaches when working with people with Wernicke's aphasia. They described developing their practice through trial and error and reported they changed their views regarding the theoretical basis for treatment over time as a result of treating individuals with Wernicke's aphasia. More research is needed to understand the reasons for this. SLTs working in stroke units and ESD teams reported delivering CPT in the acute stages post‐stroke, although the evidence base to support this currently does not exist. SLTs expressed uncertainty about which communication strategies to recommend to PwWA and their communication partners. Empirical research into interactions between PwWA, their friends, family members and healthcare professionals is also lacking. This would provide a better understanding of which communication strategies should be recommended, when and to whom.

## 5 Conclusion

Many SLTs viewed working with PwWA negatively. They wanted to help but felt unsure about how to do so, and commented that the current evidence base was limited. SLTs felt that the lack of insight into communication difficulties experienced by PwWA affected all aspects of therapy, from establishing a therapeutic relationship to goal setting, assessment and treatment. SLTs offered CPT to PwWA across the stroke pathway, starting in the acute stage post‐stroke. There was uncertainty around which communication strategies to recommend. SLTs reported that members of the MDT needed a stronger understanding of Wernicke's aphasia to enable PwWA to access their services.

## Funding

This study was funded by an NIHR Pre‐doctoral Clinical and Practitioner Academic Fellowship (reference NIHR302677) awarded to the first author. The views expressed are those of the authors and not necessarily those of the NIHR or the Department of Health and Social Care.

## Ethics Statement

The study on which the paper is based received full ethical approval from the University College London Department of Language and Cognition Ethics Committee (reference LCD‐2023‐04).

## Conflicts of Interest

The authors declare no conflicts of interest.

## Supporting information




**Supporting Information**: jlcd70299‐supp‐**0001**‐SuppMat.docx

**Supporting Information**: jlcd70299‐supp‐0002‐SuppMat.docx

**Supporting Information**: jlcd70299‐supp‐0003‐SuppMat.docx

## Data Availability

Participants did not give written consent for their data to be shared publicly, so supporting data are not available.
