# Supplementary material for: Speech and Language Therapists’ Views and Experiences of Working With People With Wernicke's Aphasia: A Qualitative Interview Study
Source: Int J Lang Commun Disord. 2026 Jul 25;61(5):e70299. doi: 10.1111/1460-6984.70299 (PMC13401223; doi:10.1111/1460-6984.70299)
Supplement: Supplementary file 1 — Supporting Information: jlcd70299‐supp‐0001‐SuppMat.docx [file JLCD-61-0-s002.docx]

**Supplementary File 1. COREQ Checklist**

| *Item* | *Section and subheading* |
| --- | --- |
| ***Domain 1: Research team and reflexivity*** | |
| *Characteristics* | |
| 1. **Interviewer/facilitator**: which author/s conducted the interviews/focus groups? | Methods, data collection and transcription |
| 2. **Credentials**: what were the researcher’s credentials e.g. PhD? | Methods, trustworthiness and bias |
| 3. **Occupation**: what was their occupation at the time of the study? | Methods, trustworthiness and bias |
| 4. **Gender**: was the researcher male or female? | Female |
| 5. **Experience and training**: what experience or training did the researcher have? | Methods, trustworthiness and bias |
| *Relationship with participants* | |
| 6. **Relationship established**: was a relationship established prior to study commencement? | No |
| 7. **Participant knowledge of the interviewer**: what did the participant know about the researcher? E.g. personal goals, reasons for doing the research | Methods, trustworthiness and bias |
| 8. **Interviewer characteristics**: what characteristics were reported about the interviewer/facilitator? E.g. Bias, assumptions, reasons and interests in the research topic | Methods, trustworthiness and bias |
| ***Domain 2: Study design*** | |
| *Theoretical framework* | |
| 9. **Methodological orientation and theory**: what methodological orientation was stated to underpin the study? E.g. grounded theory, discourse analysis, ethnomethodology, phenomenology, content analysis | Methods, trustworthiness and bias |
| *Participant selection* | |
| 10. **Sampling**: how were participants selected? E.g. purposive, convenience, consecutive, snowball | Methods, recruitment and sampling |
| 11. **Method of approach**: How were participants approached? E.g. face to face, telephone, email, mail | Methods, recruitment and sampling |
| 12. **Sample size**: How many participants were in the study? | Methods, participants |
| 13. **Non-participation**: How many people refused to participate or dropped out? Reasons? | One person could not connect to videoconferencing. Two people expressed an interest then were unable to participate. |
| *Setting* | |
| 14. **Setting of data collection**: Where was the data collected? E.g. home, clinic, workplace | Methods, data collection and transcription |
| 15. **Presence of non-participants**: Was anyone else present apart from the participants and researchers? | Two participants completed interviews from shared offices with the remaining interviews conducted in private rooms. |
| 16. **Description of sample**: What are the important characteristics of the sample? E.g. demographic data, date | Methods, participants |
| *Data collection* | |
| 17. **Interview guide**: Were questions, prompts or guides provided by the authors? Was it piloted? | Methods, topic guide |
| 18. **Repeat interviews**: Were repeat interviews carried out? If yes, how many? | No |
| 19. **Audio/visual recording**: Did the research use audio or video recording to collect the data? | Methods, data collection and transcription |
| 20. **Field notes**: Were field notes made during or after the interview or focus group? | Methods, trustworthiness and bias |
| 21. **Duration**: What was the duration of the interview or focus group? | Methods, data collection and transcription |
| 22. **Saturation**: Was data saturation discussed? | Methods, participants |
| 23. **Transcripts returned**: Were transcripts returned to participants for comment and/or correction? | Methods, data analysis |
| ***Domain 3: Analysis and findings*** | |
| *Data analysis* | |
| 24. **Number of data coders**: How may coders coded the data? | Methods, data analysis |
| 25. **Description of the coding tree**: Did authors provide a description of the coding tree? | Supplementary file 3: Thematic index |
| 26. **Derivation of themes**: Were themes identified in advance or derived from the data? | Methods, data analysis |
| 27. **Software**: What software, if applicable, was used to manage the data? | Methods, data analysis |
| 28. **Participant checking**: Did participants provide feedback on the findings? | Methods, trustworthiness and bias |
| *Reporting* | |
| 29. **Quotations presented**: Were participant quotations presented to illustrate the themes/findings? Was each quotation identified e.g. participant number? | Results, throughout |
| 30. **Data and findings consistent**: Was there consistency between the data presented and the findings? | Results and discussion, throughout |
| 31. **Clarity of major themes**: Were major themes clearly presented in the findings? | Results, throughout |
| 32. **Clarity of minor themes**: Is there a description of diverse cases or discussion of minor themes? | Results, throughout |
