# Supplementary material for: Speech and Language Therapists’ Views and Experiences of Working With People With Wernicke's Aphasia: A Qualitative Interview Study
Source: Int J Lang Commun Disord. 2026 Jul 25;61(5):e70299. doi: 10.1111/1460-6984.70299 (PMC13401223; doi:10.1111/1460-6984.70299)
Supplement: Supplementary file 2 — Supporting Information: jlcd70299‐supp‐0002‐SuppMat.docx [file JLCD-61-0-s001.docx]

## **Supplementary File 2. Topic Guide**

# **1. Introduction and context setting.**

- Introduce researcher and research topic.
- Explain confidentiality and anonymity.
- Explain recording length (approx. 1 hour), that they can withdraw at any time without a reason and do not have to answer any questions that they do not wish to.
- Check if they have any questions.
- Check if they are happy to continue.
- Explain how Wernicke’s aphasia is defined for purposes of this study:
  - fluent and well-articulated speech with normal prosody and good sentence structure but meaningless at times due to lexical retrieval difficulties,
  - auditory comprehension is affected despite normal or corrected hearing.
- In clinical practice, what would you call this type of aphasia?

# **2. Background.**

Aims: To get the participant talking, find out the context in which they are working and experience that they have working with PwWA.

- Setting/s that they are working in e.g. stroke unit, private practice etc.
- Service that they provide to PwWA:
  - Scope of the service e.g. assessment, treatment, discharge planning, counselling.
  - Limits upon number of sessions or length of involvement.
  - Members of the Multi-Disciplinary Team (MDT) they work regularly with to support PwWA.
  - Services that they work with e.g. voluntary sector, stroke groups.
- Explore how they developed their knowledge and skills in working with PwWA. Potentially through:
  - training e.g. university, courses, Clinical Excellence Networks,
  - supervision,
  - Continuing Professional Development e.g. own reading, reflection.

# **3. Success in assessment and treatment.**

Aims: To explore the factors which SLTs consider important in successful treatment for Wernicke’s aphasia.

Ask them to describe a time they assessed and treated someone with Wernicke’s aphasia, and it went well.

If not mentioned, explore further:

- rationale for assessment choice,
- rationale for treatment choice,
- support provided to friends and family,
- MDT working including MDT understanding of Wernicke’s aphasia and MDT providing reabling environment,
- insight that the person with Wernicke’s aphasia had into their difficulties,
- the nature and extent of any cognitive impairment that the person with Wernicke’s aphasia had including memory and attention to engage in intervention, carryover.

# **4. Challenges in assessment and treatment.**

Aims: To explore the challenges around delivering assessment and treatment to PwWA.

Ask them to describe a time when they worked with someone with Wernicke’s aphasia, and there were challenges.

If bullet point items listed in section 3 are not discussed, explore these further.

If they have not mentioned difficulties with engagement in therapy, go on to explore:

- whether they have ever had difficulties engaging someone with Wernicke’s aphasia in SLT,
- how they managed this.

# **5. Communication Partner Training.**

Aims: To find out if and how SLTs are using CPT with PwWA. To explore the strengths and challenges of using CPT with PwWA.

Establish whether they ever used CPT (or elements of) with someone with Wernicke’s aphasia. This could include:

- approaches such as total communication training and/or specific programmes e.g. Supporting Partners of People with Aphasia in Relationships and Conversation (SPPARC), Better Conversations,
- training dyads, family and friends, and/or healthcare professionals,
- formal or informal delivery.

Explore which aspects of CPT they have found helpful and which aspects that they have found challenging. If not covered, explore further:

- provision of general information about aphasia,
- provision of tailored information about communication strategies,
- reviewing personalised video recordings,
- homework.

If they have not used CPT, explore reasons for not using it.

# **6. Future suggestions.**

Aims: To find out what positive elements of practice SLTs should adopt with future patients with Wernicke’s aphasia and end the interview on a positive note:

- “If you were supervising a newly qualified SLT treating someone with Wernicke’s aphasia, what tips would you give them?”
- “In an ideal world, what would assessment and treatment provided by SLTs to PwWA look like?”
- “What would you need to be in place to provide the care that you want to?”
- Anything else they would like to add.

**7. End Recording**

- Thank the participant for their time.
- Explain what will happen next with transcription and analysis.
- Confirm that gift voucher for participation will be sent.
- Check whether the participant would like a copy of the results.
