# Supplementary material for: Speech and Language Therapists’ Views and Experiences of Working With People With Wernicke's Aphasia: A Qualitative Interview Study
Source: Int J Lang Commun Disord. 2026 Jul 25;61(5):e70299. doi: 10.1111/1460-6984.70299 (PMC13401223; doi:10.1111/1460-6984.70299)
Supplement: Supplementary file 3 — Supporting Information: jlcd70299‐supp‐0003‐SuppMat.docx [file JLCD-61-0-s003.docx]

**Supplementary File 3. Thematic Index**

1. Background information
   1. SLTs background
   2. Service description and capacity
   3. Service MDT make up
   4. Case history information for positive case
   5. Case history information for challenging case
   6. Other
2. Assessment
   1. Identification of Wernicke’s aphasia
   2. Formal language assessment
   3. Informal language assessment
   4. Observation based assessment
   5. Identifying effective communication strategies
   6. Other
3. Treatment
   1. Intervention targeting impairment
   2. Intervention targeting activity and participation
   3. Low tech AAC
   4. Groups and peer support
   5. Measuring outcomes
   6. Timing, intensity and delivery of intervention
   7. Other
4. Working with the person with Wernicke’s aphasia
   1. Goals
   2. Wider disabilities
   3. Cognition
   4. Insight
   5. Quality of life and mental wellbeing
   6. Engagement and relationship building
   7. Aphasia education
   8. Decision for discharge from SLT or rehab service
   9. Other
5. Working with family and friends
   1. Barriers
   2. Facilitators
   3. Carers expectations
   4. Carers mental wellbeing
   5. SLT support for carers
   6. Support from other professionals for carers
   7. Aphasia education
   8. Other
6. Communication partner training
   1. Approaches to CPT
   2. Modelling
   3. Video recording
   4. Facilitators of CPT
   5. Barriers to CPT
   6. Other
7. Working with the MDT
   1. MDT joint working
   2. Mental capacity assessment
   3. Discharge to permanent place of residence
   4. MDT training
   5. Other
8. Professionals knowledge, skills and attitudes
   1. SLT university training and NQPs resulting knowledge and skills
   2. SLT development of knowledge and skills working with PwWA
   3. SLT attitudes and feelings towards working with PwWA
   4. Theoretical framework for Wernicke’s aphasia
   5. Labelling Wernicke’s aphasia
   6. MDT knowledge and skills working with PwWA
   7. Attitudes and feelings of other health and social care professionals towards working with PwWA
   8. Other
9. Other
